# A Novel Type of Autosomal Dominant Episodic Nystagmus Segregating with a Variant in the FRMD5 Gene

**DOI:** 10.1080/01658107.2024.2338562

**Published:** 2024-04-22

**Authors:** Björn Hammar, Sofia Paulsson, Hafdis T. Helgadottir, John Albinsson, Magdalena Naumovska, Rafi Sheikh, Malin Kvarnung

**Affiliations:** a Department of Ophthalmology, Department of Clinical Sciences Lund, Lund University, Skåne University Hospital, Lund, Sweden; b Department of Ophthalmology, County Hospital Ryhov, Jönköping, Sweden; c Department of Molecular Medicine and Surgery, Karolinska Institutet, Stockholm, Sweden; d Department of Clinical Genetics, Karolinska University Hospital, Stockholm, Sweden

**Keywords:** Hereditary, autosomal dominant, nystagmus, episodic, *FRMD5*

## Abstract

To describe the phenotype of a novel form of autosomal dominant episodic nystagmus and to identify the potential genetic aetiology. We identified several individuals in a large Swedish family affected by episodic nystagmus. In total, 39 family members from five generations were invited to participate in the study, of which 17 were included (12 affected and 5 unaffected). The phenotype of the nystagmus was described based on data collected from family members through questionnaires, interviews, clinical examinations and from video recordings of ongoing episodes of nystagmus. Whole genome sequencing (WGS) and further Sanger sequencing for segregation of the identified candidate variants was performed in eight participants (six affected and two unaffected). The 12 affected participants showed a phenotype with episodic nystagmus of early onset. A vertical jerk nystagmus with variable amplitude and frequency was characterized in the analysed video material. No other eye pathology or other disease that could explain the episodic nystagmus was identified among the family participants. Genetic analysis identified a missense variant (p.Ser375Phe) in the gene FRMD5, which segregated with the disease in the eight individuals analysed, from three generations. We describe a novel autosomal dominant form of early onset episodic nystagmus and suggest the FRMD5 gene as a strong candidate gene for this disorder.

## Introduction

Nystagmus is defined as an oscillating repetitive movement of the eye that can occur either as a physiological or as a pathological phenomenon.^1^ The visual consequences of nystagmus include visual impairment and oscillopsia due to motion of the retinal image. In addition to reduced visual acuity, nystagmus can also have negative effects on the quality of life.^2^

Several subgroups of nystagmus have been described based on their clinical appearance. Pathological forms of nystagmus can arise from a wide range of disorders, such as diseases of the vestibular system, neurological diseases, disorders of the afferent pathways of the eye or they may be idiopathic.^1^ Depending on the age of onset, nystagmus can be classified as either acquired or infantile.

Genetic forms of nystagmus, associated, for example, with oculocutaneous albinism, Leber’s congenital amaurosis, optic hypoplasia or aniridia, can result from constitutional mutations in genes affecting the embryogenesis of the visual system.^3–8^ Among patients with infantile nystagmus, with otherwise normal findings on eye examination and no known eye pathology, the *FMRD7* gene has been identified as the major cause of X-linked idiopathic congenital nystagmus.^9^

Another subset of inherited disorders associated with nystagmus are episodic ataxias (EA), a group of autosomal dominant channelopathies characterized by sudden episodes of ataxia associated with other accompanying symptoms such as vertigo, nausea, and weakness. Episodes of ataxia are often triggered by emotional stress and physical exercise.^10^ Apart from EA, to the best of our knowledge, few hereditary forms of episodic nystagmus have been described in otherwise healthy individuals and none which has an established genotype.

The aim of this study was to describe the phenotype of a novel form of hereditary episodic nystagmus seen in a Swedish family, and to perform genome sequencing to identify the potential genetic aetiology.

## Materials and methods

### Subjects

**Figure 1.**
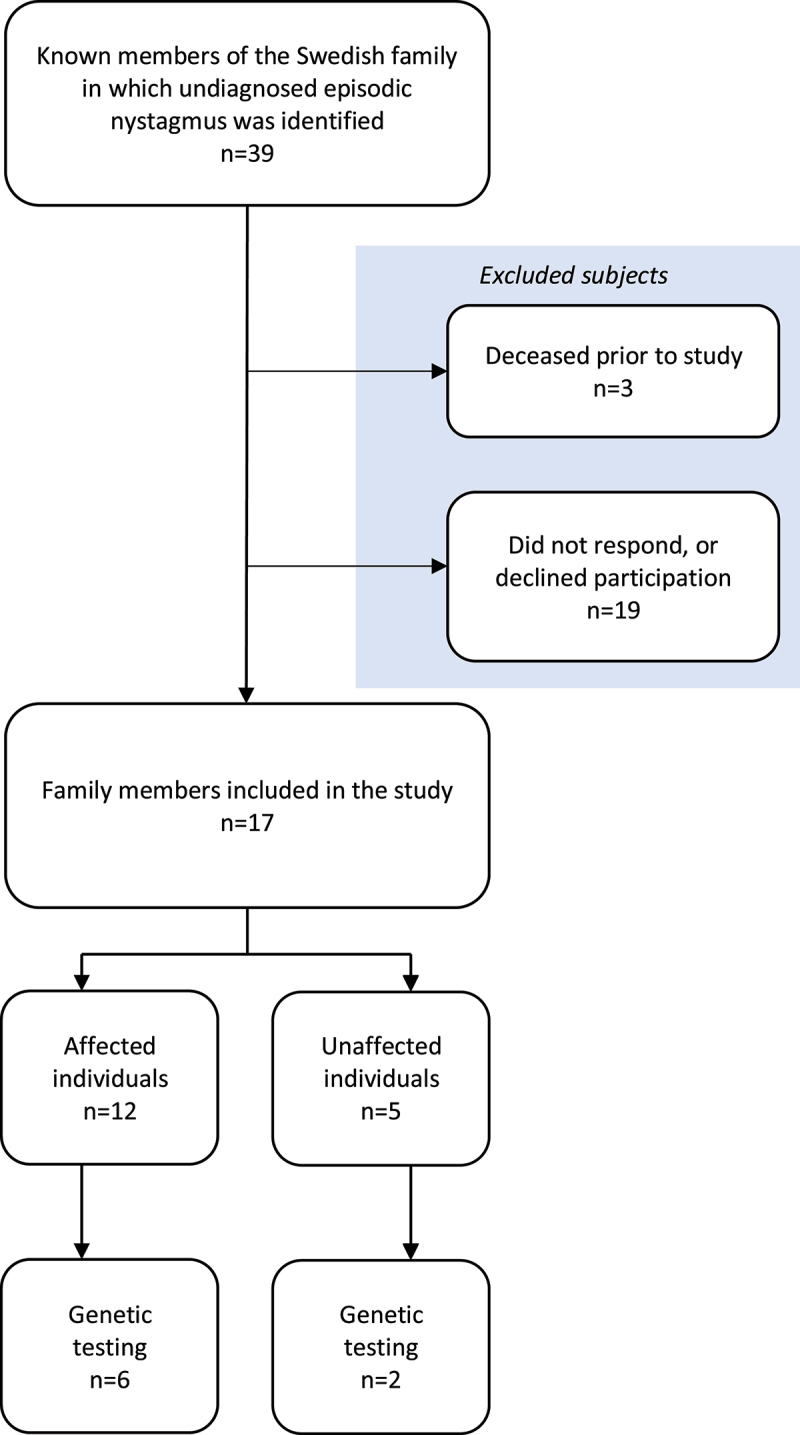
Flowchart showing enrolment of participants.

The participants of this study were all recruited from the same Swedish family. In total, 39 individuals from five generations were included in the pedigree, of whom 18 were affected and 21 unaffected. All living family members were invited to take part in the study. Of the affected individuals, 16 were alive at the start of this study, and 12 of them were willing to participate. Five unaffected individuals also took part in the study. The remaining family members declined participation in the study. A flowchart showing the enrolment of subjects is shown in Figure 1.

Seven of the affected participants had previously undergone neuroimaging (computed tomography or magnetic resonance imaging) showing no pathological findings of significance that could cause nystagmus. One of the affected participants had undergone an electroencephalogram, which showed normal results. Four of the affected participants had undergone clinical neurological examination, with normal results. Four of the affected participants had undergone ophthalmological examination for other reasons prior to the study, three of them showing normal results and one who was diagnosed with glaucoma and had been surgically treated for amotio retinae and cataract. No significant eye diseases that would cause nystagmus were seen. There were no reports of strabismus. Five of the affected participants had Thomsen disease (myotonia congenita), and four reported having migraine. One had previously undergone surgery for a meningioma. The characteristics of the 12 affected participants are given in Table 1.

**Table 1.** Characteristics of the 12 affected participants. Data obtained from questionnaires and medical records.

	Affected participants (*n* = 12)
Gender (male/female)	5/7
Median age (years) (range)	51.5 (13–81)
Median age at onset (years) (range)	2.5 (0.5–8)
*Other diagnoses/conditions*	
Epilepsy (n)	0
Migraine (n)	4
Other neurological disease (n)	0
Psychiatric disease (n)	1
Myotonia congenita (n)	5
Strabismus (n)	0
Previous neuroradiology examination with normal results (n)	7
Previous electroencephalogram with normal results (n)	1
Previous clinical neurological examination with normal results (n)	4

## Ethics

The study was approved by the Swedish Ethical Review Authority. The research adhered to the Declaration of Helsinki as amended in 2008. Informed consent was obtained from all participants, after being given verbal and written information about the study.

### Clinical data collection

Clinical data were collected using a questionnaire that was sent to all the participating family members, in which they were asked about episodes of oscillating eye movements, details of the episodes, age of onset, intensity over time, number and frequency of episodes, precipitating or alleviating factors and previous therapeutic attempts. Additional data regarding their medical history, i.e. the results of previous neuroradiology, and ophthalmological and neurological examinations, were obtained from medical records.

The affected participants were invited to a neuro-ophthalmological examination at Skåne University Hospital in Lund, Sweden. Six of them accepted and were examined. The examination included best-corrected visual acuity, pupillary response, range of eye movements, pursuit, and saccadic eye movements. Any nystagmus was noted. The anterior parts of the eye and the fundus were examined using a slit lamp biomicroscope. During the visit, these six affected participants were also interviewed in-depth about their symptoms to obtain a thorough understanding of the disorder.

### Genetic analysis

**Figure 2.**
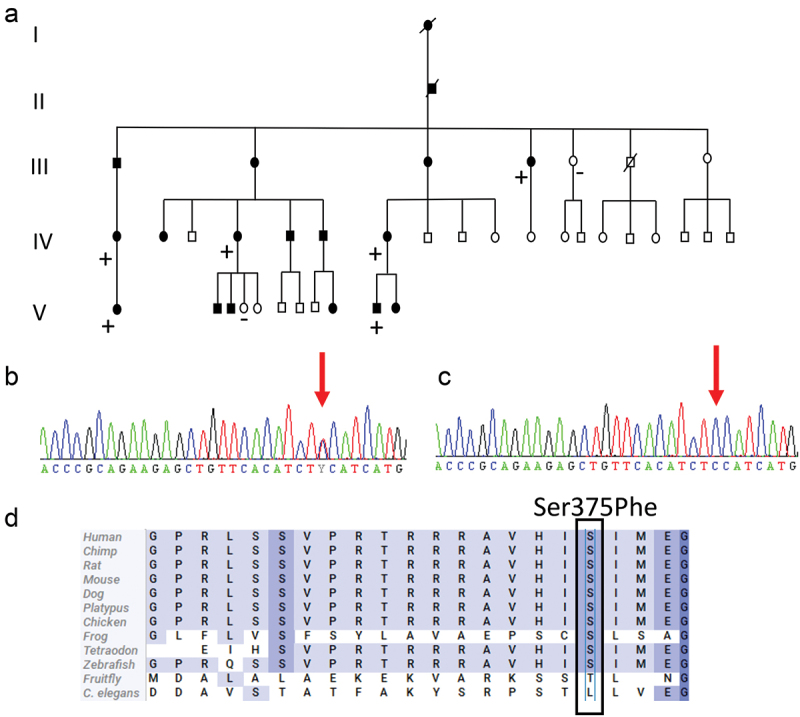
(a) Pedigree of the family with a Ser375Phe (c.1124C>T) variant in the *FRMD5* gene. Plus and minus indicate individuals with and without the variant, respectively. Squares: male; circles: female; slash: deceased. Filled symbols indicates affected family members and unfilled healthy family members. (b) Chromatogram of a heterozygous carrier of a C to T change at nucleotide 1124 of *FRMD5* (NM_032892). (c) Chromatogram showing a wild-type sequence in nucleotide 1124 of *FRMD5* (NM_032892). (d) Alignment of a partial sequence of human FRMD5 protein with 12 protein sequences.

All the participants were asked to give blood samples for DNA analysis; six affected and two unaffected agreed to do so. DNA was then extracted from the blood samples of these eight family members. Samples from four affected participants were analysed with genome sequencing (GS), and further segregation for the identified candidate variants was determined by Sanger sequencing in the additional four samples (two affected and two unaffected) (Figure 2). In GS, extracted DNA was converted to sequencing libraries using a polymerase chain reaction-free paired-end protocol (Illumina TruSeq DNA PCR-free), and sequencing was performed using the Illumina NovaSeq 6000 platform with 30× median coverage. Sequencing data were processed using a combination of pre-existing and custom-developed open source tools for the detection of single nucleotide variants, small insertions and deletions, structural variants and repeat expansions, as described previously.^11^ To identify potential candidate variants, the data were filtered for intragenic, rare (minor allele frequency <0.1%) variants that were shared between the four affected individuals.

### Video analysis of eye movements

The affected family members participating in the study were instructed to record any episodes of nystagmus using their smart phones to provide documentation of eye movements. The length of the video was required to be at least 30 seconds to obtain sufficient material to analyse.

The videos were loaded into Matlab (The MathWorks Inc., Natick, MA, USA) for analysis to estimate the position of the cornea. A speckle tracking method^12,13^ was used to estimate the relative motion of the head of the individual. An area around each eye was selected using the estimated motion of the head, thus minimizing the influence of head and camera motion during video recording. The pixel values in the selected area were limited to a certain threshold, to minimize the presence of any reflections in the eye from ambient lighting. The extracted image areas were high-pass filtered to highlight the border of the cornea. The border of the cornea was again tracked using the same speckle tracking method, giving a total motion (the motion of the cornea plus a global motion). From this, the motion of the cornea was calculated as the difference between the total motion and the global motion. The frequency of the motion of the cornea was estimated using the Fourier transform of the motion data, and verified by visual inspection of the video sequence.

## Results

### Clinical characteristics

**Figure 3.**
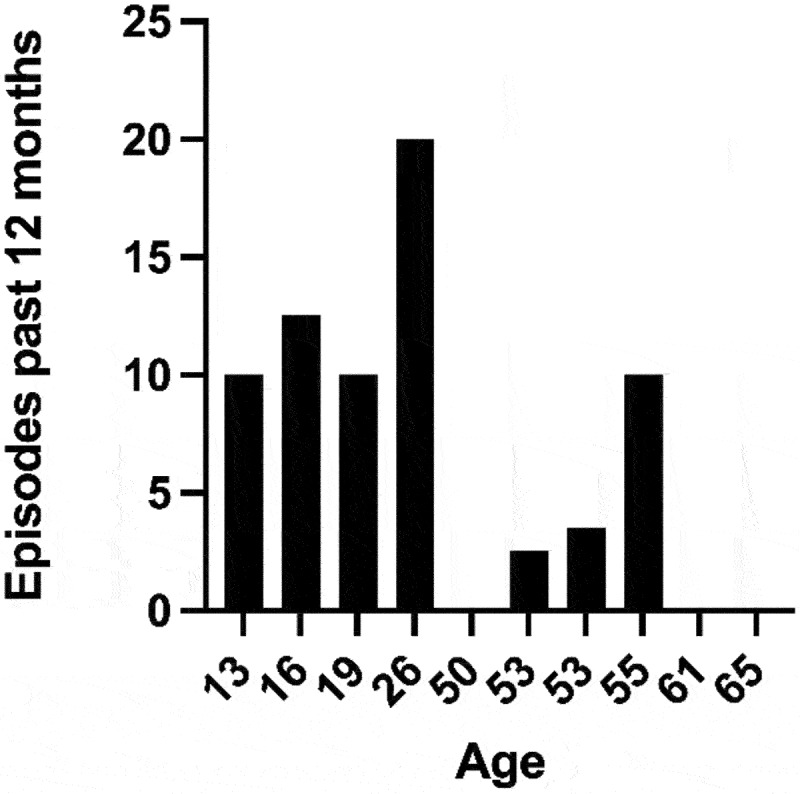
Number of episodes of nystagmus during the last 12 months, reported by each of the affected participants. Some subjects gave a range for the number of episodes, in which case, the mean value was used.

All the affected family members participating in the study reported oscillating eye movements. This was confirmed by analysis of video recordings provided by three of the affected participants. The age of onset varied from six months to eight years. There was a considerable difference in the reported number of episodes during the 12 months preceding the examination, ranging from none up to 20. Adolescence and the early twenties were generally described as the most active period of the disease. Most participants had few active episodes of disease past the age of 50, although one reported a decline in the number of episodes as early as the age of 30–35 years. More episodes were reported among young participants (Spearman correlation *r* = −0,67, *p* = <0,05) (Figure 3). Table 2 presents details of the clinical characteristics of nystagmus episodes in the 12 affected participants.

**Table 2.** Characteristics of nystagmus episodes in the 12 affected participants, obtained from questionnaires. (Some data are missing for three participants since they did not respond to all questions. When subjects answered a question with a range of numbers, the mean of the range was used.).

Median age at onset (range) (years)	2.5 (0.5–8)
Median number of episodes in the past year (range)	6.75 (0–20)
Median maximal number of episodes per day (range)	1 (0–3)
Median minimal number of episodes per day (range)	0 (0–1)
Median age when frequency of episodes decreased^1^ (range)(years)	50 (32.5–53)
Median age when frequency of episodes reached a maximum (range)(years)	20.25 (1–50)

^1^The four youngest subjects (aged 13–26) reported that the number had not yet decreased, and these data were excluded.

Associated symptoms were also reported during episodes of nystagmus by most of the participants. The most commonly reported symptoms during an episode were diplopia, nausea, and oscillopsia, while poor balance, vertigo, and impaired vision were also reported. Among the factors that could provoke an episode, bright light was the most common, but physical and emotional stress were also reported as precipitating factors. Different approaches were adopted to alleviate the symptoms resulting from the nystagmus, such as resting, closing the eyes and looking downwards. Details concerning associated symptoms, precipitating factors and alleviating factors can be found in Table 3.

**Table 3.** Percentage of the total number of affected subjects (*n* = 12) reporting associated symptoms during ongoing episodes of nystagmus, precipitating factors that could trigger an episode, and alleviating factors that reduced the severity and number of nystagmus episodes.

	%
**Associated symptoms**	
Diplopia	92
Nausea	83
Oscillopsia	83
Poor balance	75
Vertigo	75
Impaired vision	75
Fixed gaze	50
Vomiting	17
**Precipitating factors**	
Bright light	92
Strenuous activity	83
Emotional stress	75
Tiredness	67
Hunger	50
Crowded places	50
Strong wind	50
Sweating	42
Strong colours	33
Specific patterns	25
Caffeine	0
**Alleviating factors**	
Rest	100
Closing the eyes	83
Looking downwards	75
Avoiding stress	58
Eating regularly	50
Shielding from the sun with a cap or sunglasses	42
Gaze fixation	17

Further details on the characteristics of episodes of nystagmus among the affected participants were obtained from interviews during visit to the clinic. All the interviewed participants reported that episodes of nystagmus had considerable impact on the performance of their daily activities, and that they avoided activities that could trigger these episodes. Some required rest or a calm environment to relieve their symptoms, while closing their eyes for a while was sufficient for others. The length of the episodes varied from a few minutes up to approximately an hour. Alcohol was also mentioned as a trigger, especially when consumed in busy, crowded places, such as a discotheque or a bar. Many of the interviewed participants reported that situations requiring full concentration in combination with physical stress triggered episodes of nystagmus. Examples of these situations were competitive horse jumping, ball sports, and cycling against the clock. Situations that required only mental focus, such as sitting an examination in school, did not normally provoke nystagmus.

The affected participants reported few examples of efficient medical therapies, despite attempts with various medications over the years. Gabapentin was reported by one of the participants to have a transient effect in reducing vertigo and nausea, although the treatment was later discontinued due to the decrease in the number of episodes with age. Two of affected participants had been recommended paracetamol as a prophylaxis for situations that normally triggered an episode, although it was not clear whether this had any effect.

### Motility characteristics

**Figure 4.**
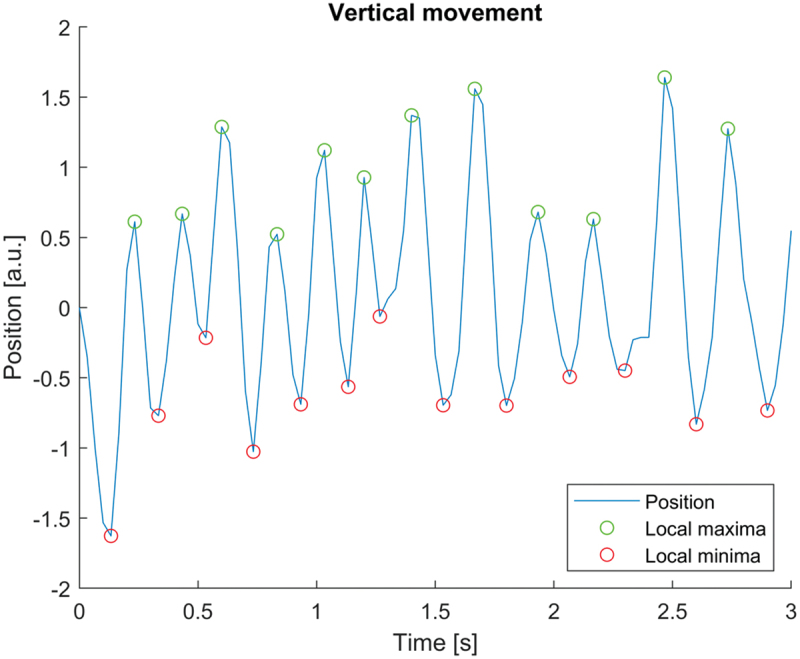
Analysis of video recordings from an affected family member, showing an upbeat nystagmus with variable amplitude and frequency.

A majority of affected participants reported changes in eye movement during episodes of nystagmus that led to a reduction in vision. Three of the participants were able to record an ongoing episode. A vertical jerk nystagmus with variable amplitude and frequency was seen in the analysed video material. Two affected participants had upbeat nystagmus, and one had a downbeat nystagmus. No periods of foveation were observed. Intervals with higher amplitude and lower frequency, around 2 Hz, as well as intervals with lower amplitude and higher frequency, around 5 Hz, were observed (Figure 4). Lid nystagmus was also observed, in the form of synchronous movements of the upper eyelids together with the upbeat eye movements.

Attempts had been made before the start of this study to provoke an episode of nystagmus in a clinically controlled setting, but without success. However, an episode of nystagmus occurred in one participant during the clinical examination when testing their range of eye movements. An vertical nystagmus was seen in all gaze directions, except downwards, where a null point was identified.

### Genetic results

Two candidate variants were identified by WGS, a transition in the 3′ UTR region of the *ATXN1* gene (c. *5365T>C) and a missense variant in the *FRMD5* gene (NM_032892.5 c.1124C>T, p.Ser375Phe). However, after segregation, only the variant in the *FRMD5* gene remained as a potential pathogenic variant. The c.1124C>T variant in *FRMD5* and its segregation in the family is shown in Figure 2a-c. The *FRMD5* variant is absent in the general population, according to gnomAD,^14^ and was predicted to be deleterious or damaging by PolyPhen^15^ and SIFT.^16^ The Combined Annotation Dependent Depletion score for the variant was 25.^17^ Furthermore, the serine residue in position 375 was highly conserved, as shown in Figure 2d.

## Discussion

This study describes a large family from southern Sweden, with a previously undefined autosomal dominant form of early onset episodic nystagmus that segregates with a missense variant in *FRMD5*. The nystagmus was identified as a vertical jerk nystagmus, of variable amplitude without foveation periods. Vertical jerk nystagmus is most often seen as a result of an acquired lesion to the brainstem, pons, or midbrain. It has also been noted in patients with Wernicke’s encephalopathy.^18,19^ Neuroimaging and clinical examination provided no evidence of acquired structural damage in the central nervous system that could lead to nystagmus among the affected family members participating in this study.

When reviewing the medical background of the family it was discovered that five of the family members had previously been diagnosed with Thomsen disease, an autosomal dominant disorder causing muscle stiffness. Thomsen disease, also known as myotonia congenita, is the result of a mutation in a chloride channel in the muscle cell membrane.^20^ Myotonia congenita has been associated with various eye-related conditions such as strabismus, ptosis, hypertrophy of extraocular muscles, and slow saccades,^21–23^ However, to the best of our knowledge, there is no evidence that myotonia congenita causes nystagmus. The five individuals with myotonia congenita all came from the same branch of the family tree, and as far as they knew, the condition had been inherited from a person not involved in the pedigree of the family examined in this study. No genetic analysis had been carried out to confirm this diagnosis. Furthermore, none of the other family members in the other branches has been diagnosed with myotonia congenita, and they did not complain of any muscle stiffness. We therefore believe that myotonia congenita is unlikely to be related to the episodic nystagmus investigated in this study, although it is remarkable that five of the individuals exhibited two different, very rare inherited disorders.

Considering that horizontal oscillations are most frequently seen in hereditary congenital forms of nystagmus, our finding of vertical oscillations is unusual. Familial forms of congenital vertical nystagmus are uncommon.^24,25^ Episodic ataxia type 2 (EA2) is a subset of inherited disorders that presents with vertical nystagmus. EA2 has been linked to mutations in the *CACNA1A* gene, encoding a calcium channel complex that is abundant in the central nervous system and the cerebellum.^10,26^ EA2 was considered as a differential diagnosis for the family described in this study due to similarities in phenotype, such as the mode of inheritance, the episodic nature of nystagmus, and the resemblance of accompanying symptoms. However, apart from nystagmus, we have not identified any other signs of impaired coordination of voluntary muscle movement, which is a key feature in patients with EA2.^27^ Furthermore, no genetic variants were detected in the CACNA1A gene by WGS in the present study.

Only a few cases of hereditary vertical nystagmus in otherwise healthy individuals could be found in the literature: for example, an Italian family of five generations with early onset of a vertical pendular nystagmus without signs of ataxia,^28^ downbeat nystagmus seen in an eight-year-old boy and his mother,^29^ and a congenital vertical pendular nystagmus in two sisters.^30^ There are also descriptions of families with inherited forms of nystagmus that present with combined vertical and horizontal nystagmus, and some of the families also present with mild ataxia.^24,31^ Interestingly, Sogg and Hoyt^32^ described a father and son with intermittent upbeat vertical nystagmus of early onset and a clinical picture almost identical to that seen in our Swedish family. The precipitation of attacks by fatigue and emotional stress, blurred vision and oscillopsia during attacks, and improvement when looking downwards with the head tilted backwards, are some of the shared characteristics. Moreover, Sogg and Hoyt also noted synchronous blinking with each upbeat of the eyes, corresponding to the eyelid nystagmus seen in the Swedish family. Sogg and Hoyt could not determine the mode of inheritance due to the small number of individuals, but they suggested in their case report that there was an inherent supranuclear derangement in the brainstem.^32^ In the case reports mentioned above, the hereditary vertical nystagmus is well described phenotypically, but the specific genetic aetiology was not determined.

In our study, a variant in the gene *FRMD5* (p.Ser375Phe) was identified. The variant segregates with the disorder in all of the eight analysed individuals and the *FRMD5* gene is thus considered as a candidate gene for the phenotype in the family. Pathogenicity of the variant is supported by bioinformatic prediction scores, a high degree of conservation among species, and its absence in the general population.

*FRMD5* encodes a 570-amino acid‐protein that contains an N‐terminal FERM domain and is a member of the FERM (4.1/ezrin/radixin/moesin) protein family.^33^ *FRMD5* is highly expressed in retinal bipolar c-10 ganglion cells^34^ which have been shown to be the origin of nystagmus in congenital stationary night blindness.^35^ However, the detailed biological function of *FRMD5* is largely unknown. The FERM protein family includes a large number of proteins and, interestingly, another member of this protein family, *FRMD7*, is associated with nystagmus. Mutations in the *FRMD7* gene are the cause of *FRMD7*-related infantile nystagmus (FIN), which is an X-linked disorder characterized by either the onset of horizontal, conjugate, gaze-dependent nystagmus in the first six months of life, or periodic alternating nystagmus.^36^ Patients with FIN often present with structurally normal eyes, good visual acuity, and normal colour vision.^37^

Despite differences regarding age of onset, mode of inheritance and different beating direction of the nystagmus seen in our patients and patients with FIN, it is notable that mutations in a gene that is similar to *FRMD5* are associated with a phenotype that resembles the phenotype in our patients.

It is also highly interesting that heterozygous mutations in *FRMD5* were recently linked to a novel neurodevelopmental disorder with abnormal eye movements.^38^ Lu et al. reported eight individuals with missense variants in *FRMD5* (summarized in Table S1), six of which had abnormal eye movements, described as an unspecified type of nystagmus and/or opsoclonus. The direction of the nystagmus was specified in only one of the patients, and in this case, the direction was vertical, as in our patients. The similarity between the ophthalmologic phenotype in our patients and the patients described by Lu et al. is striking. The genetic and phenotypic overlap strongly supports that *FRMD5* is the causative gene linked to nystagmus in our patients. However, the patients studied by Lu et al. also had intellectual disability, ataxia, and epilepsy, in addition to abnormal eye movements. The Ser375Phe variant reported here, as well as most of the missense variants reported by Lu et al., are located in a region where protein function is unclear and model confidence is very low (supplementary Figure S1). Therefore, we can only speculate that the Ser375Phe variant might be less deleterious than the variants reported by Lu et al., or that the affected residue specifically targets pathways that are involved in eye movements.

Due to the episodic nature of the nystagmus, characterization of the nystagmus in a clinical setting was difficult. However, the possibility of analysing video recordings of ongoing episodes facilitated and strengthened the process of describing this new form of episodic nystagmus. It would have been desirable to have video recordings from all the affected participants in our study. However, some of them did not have suitable equipment, or found it difficult to handle the equipment during ongoing episodes. DNA analysis of all the participants would have been preferable, but only eight of them agreed to undergo genetic testing.

Taken together, our results strongly suggest that *FRMD5* is the causative gene in this novel type of early onset autosomal dominant episodic nystagmus, characterized by episodes of vertical jerk nystagmus. Future functional studies and evaluations of additional patients with variants in *FRMD5* will be essential to fully establish genotype-phenotype causality.

## Conclusion

We describe a novel autosomal dominant form of early onset episodic nystagmus that is likely caused by a missense mutation in *FRMD5*.

## Supplementary Material

Supplemental Table 1

Supplemental Figure 1
