# Supplementary material for: A Novel Type of Autosomal Dominant Episodic Nystagmus Segregating with a Variant in the FRMD5 Gene
Source: Neuroophthalmology. 2024 Apr 22;48(6):407–16. doi: 10.1080/01658107.2024.2338562 (PMC11581191; doi:10.1080/01658107.2024.2338562)
Supplement: Supplemental Table 1 [file IOPH_A_2338562_SM5958.docx]

Table S1.

| **Patients/families** | ***FRMD5* variant**  **NM_032892.5** | **FRMD5 protein effect** | **Ophthalmological phenotype** | **Additional phenotype** |
| --- | --- | --- | --- | --- |
| Lu et al 1 | c.340T>C | p.Phe114Leu | strabismus | NDD, E, A, S |
| Lu et al 2 | c.1051A>G | p.Ser351Gly | nystagmus | NDD, E, A, H, S |
| Lu et al 3 | c.1053C>G | p.Ser351Arg | opsoclonus | NDD, E, A |
| Lu et al 4 | c.1054T>C | p.Cys352Arg | ocular vertical nystagmus/flutter | NDD, E, A, H, S |
| Lu et al 5 | c.1054T>C | p.Cys352Arg | nystagmus, intermittent flutter with poor fixation, mild myopia, delayed visual maturation | NDD, A, H |
| Lu et al 6 | c.1060T>C | p.Ser354Pro | opsoclonus, hypermetropia, visually impaired | NDD, A, H |
| Lu et al 7 | c.1045A>C | p.Ser349Arg | nystagmus and opsoclonus | NDD, E, A |
| Lu et al 8 | c.1637A>G | p.Tyr546Cys | intermittent esotropia | NDD, E, H, S |
| This study | c.1124C>T | p.Ser375Phe | vertical intermittent nystagmus | - |

NDD, neurodevelopmental disorder; E, epilepsy; A, ataxia; H, hypotonia; S, spasticity
