# Supplementary material for: A Novel Type of Autosomal Dominant Episodic Nystagmus Segregating with a Variant in the FRMD5 Gene
Source: Neuroophthalmology. 2024 Apr 22;48(6):407–16. doi: 10.1080/01658107.2024.2338562 (PMC11581191; doi:10.1080/01658107.2024.2338562)
Supplement: Supplemental Figure 1 [file IOPH_A_2338562_SM5719.pdf]

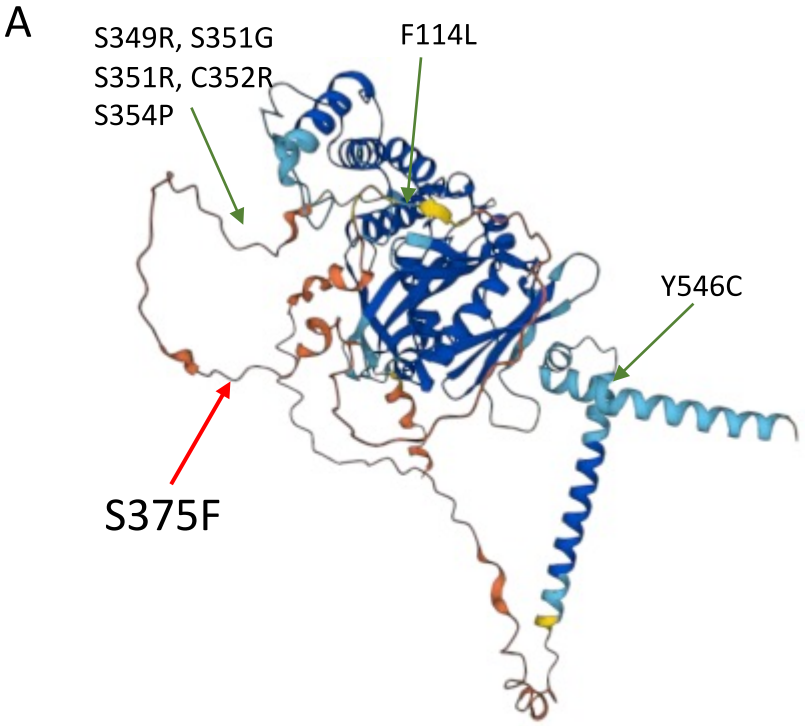

Model Confidence

Very high (pLDDT > 90)

High (90 > pLDDT > 70)

Low (70 > pLDDT > 50)

Very low (pLDDT < 50)

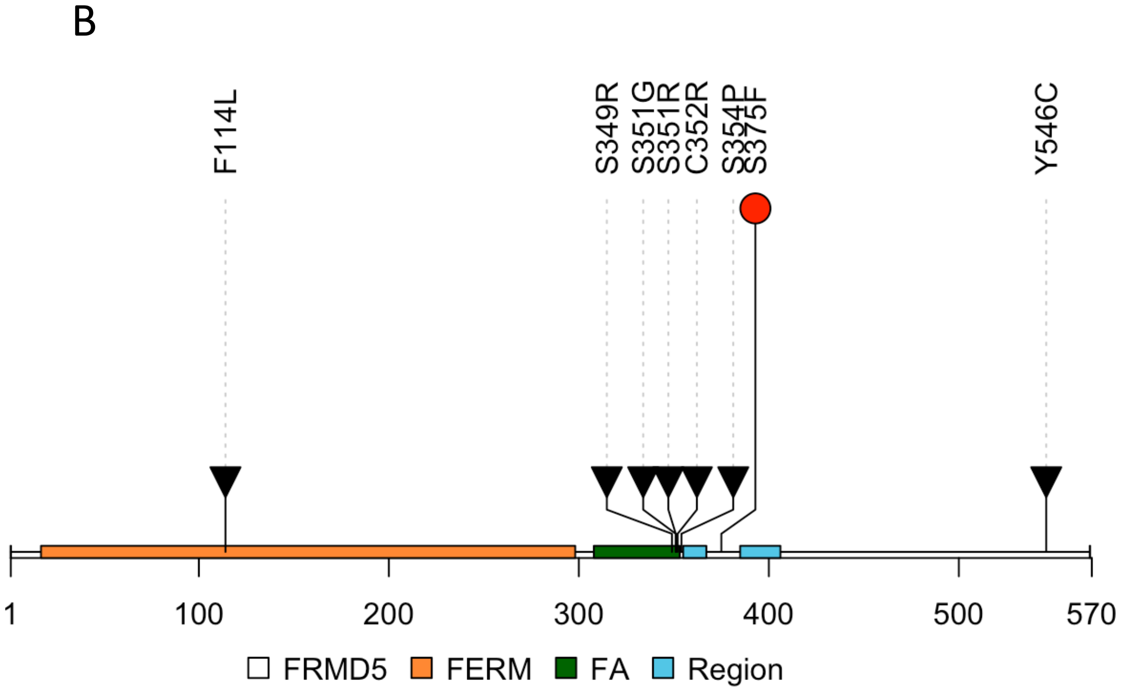

A. Predicted structure of FRMD5 reference based on AlphaFold top predicted models. The variant reported here as well as five of 7 missense reported by Lu *et al* 2022 are located in a loop that has a very low model confidence (pLDDT<50)

B. Lollipop plot showing the location of the missense variant reported in this study (red circle) as well as the variants reported by Lu *et al* 2022 (black down pointing triancels). Domain prediction based on UniProt (Q7Z6J6 FRMD5_human), FA: FERM associated domain. Region: intrinsically disordered region that lack fixed or ordered 3-D structure.
